# Air pollution and public health: emerging hazards and improved understanding of risk

**DOI:** 10.1007/s10653-015-9720-1

**Published:** 2015-06-04

**Authors:** Frank J. Kelly, Julia C. Fussell

**Affiliations:** NIHR Health Protection Research Unit in Health Impact of Environmental Hazards, MRC-PHE Centre for Environment and Health, Facility of Life Sciences and Medicine, King’s College London, 150 Stamford Street, London, SE1 9NH UK

**Keywords:** PM toxicity, Emerging risks, Public awareness, Air quality communication

## Abstract

Despite past improvements in air quality, very large parts of the population in urban areas breathe air that does not meet European standards let alone the health-based World Health Organisation Air Quality Guidelines. Over the last 10 years, there has been a substantial increase in findings that particulate matter (PM) air pollution is not only exerting a greater impact on established health endpoints, but is also associated with a broader number of disease outcomes. Data strongly suggest that effects have no threshold within the studied range of ambient concentrations, can occur at levels close to PM_2.5_ background concentrations and that they follow a mostly linear concentration–response function. Having firmly established this significant public health problem, there has been an enormous effort to identify what it is in ambient PM that affects health and to understand the underlying biological basis of toxicity by identifying mechanistic pathways—information that in turn will inform policy makers how best to legislate for cleaner air. Another intervention in moving towards a healthier environment depends upon the achieving the right public attitude and behaviour by the use of optimal air pollution monitoring, forecasting and reporting that exploits increasingly sophisticated information systems. Improving air quality is a considerable but not an intractable challenge. Translating the correct scientific evidence into bold, realistic and effective policies undisputedly has the potential to reduce air pollution so that it no longer poses a damaging and costly toll on public health.

## Introduction

### Historical perspective

Air pollution is now fully acknowledged to be a significant public health problem, responsible for a growing range of health effects that are well documented from the results of an extensive research effort conducted in many regions of the world. Whilst there is no doubt that rapid urbanisation means that we are now exposed to unhealthy concentrations and a more diverse variety of ambient air pollutants, palaeopathological research suggests the problem, in the form of smoke, plagued our oldest ancestors. Computerised tomography imaging studies on the bodies of ancient mummies have detected evidence of pneumonia, emphysema, pulmonary oedema and atherosclerosis (Zweifel et al. 2009; Thompson et al. 2013), whilst autopsies have described extensive carbon deposits in the lung (Zimmerman et al. 1971). This in turn has led to a speculative link to the daily inhalation of smoke in confined spaces from fuels used for warmth, cooking and lighting.

**Fig. 1:**
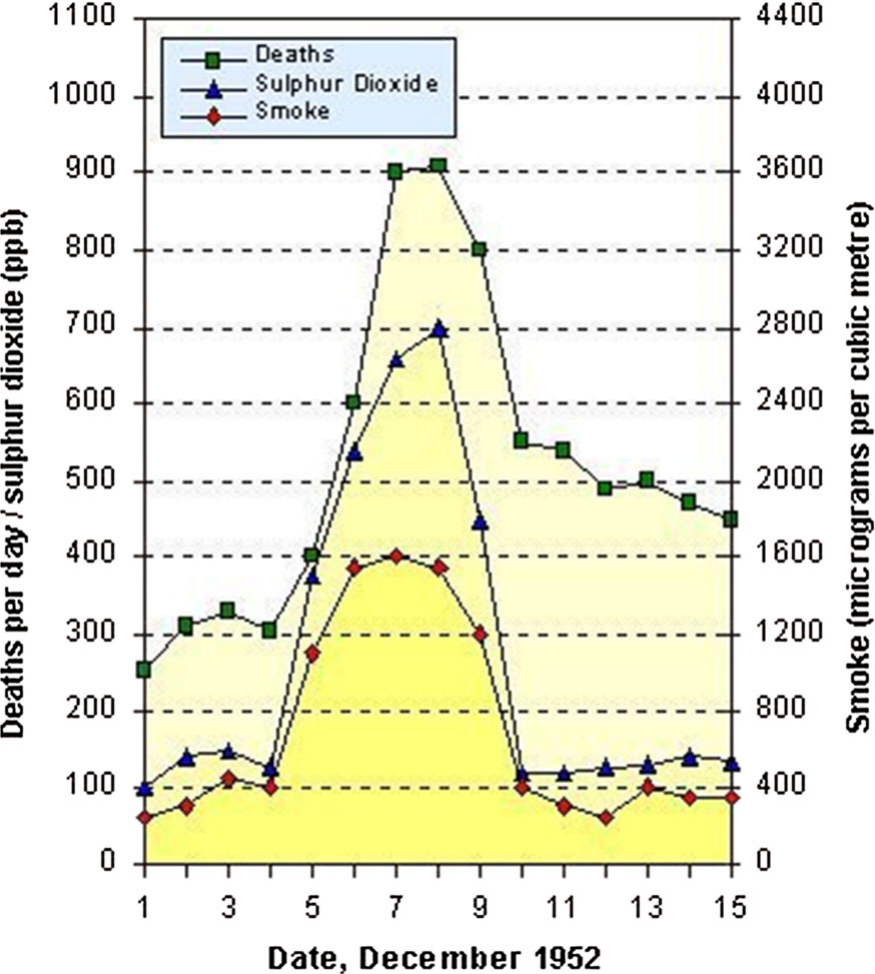
Death toll and pollution concentrations during the 1952 London Smog. *Source*: Wilkins (1954)

Leaping forward through history to Victorian London, the billowing smoke and sulphur dioxide (SO_2_) from domestic and industrial coal burning, mixed with natural fog, famously caught the imagination of literary and visual artists. They regarded this meteorological phenomenon as a spectacular manifestation of turn-of-the-century life in a cosmopolitan city. Indeed, the unique style that Charles Dickens adopted in his description of the fogs meant that they became a romantic legend. For Claude Monet, the chromatic atmospheric effects created by the effects of smog on sunlight gave London magnificent breadth and became the predominant theme in his renditions of the city. As a consequence, to some, London’s notoriously toxic air became a world-famous institution rather than an appalling social evil. In December 1952, however, a vast and lethal smog, caused by cold stagnant weather conditions that trapped combustion products at ground level, brought about the worst air pollution disaster in history, resulting in an estimated 4000–12,000 deaths and an enormous increase in respiratory and cardiovascular complications (Logan 1953; Bell and Davis 2001). This crisis was also the direct incentive to pass the Clean Air Act in 1956, which successfully curtailed domestic coal burning in London and other major cities in the UK. At this point, the UK led the world in cleaning up air by implementing smokeless zones, imposing controls on industry, increasing the availability and use of natural gas and changing the industrial and economic structure of the country. The results were considerable reductions in the concentration of smoke and SO_2_ (Wilkins 1954; Fig. 1).

### Modern-day air pollution

On recounting such progress, it is especially disappointing that in recent years, improvements in air quality, not solely within the UK but in many urban areas around the world, have miserably stalled. We occasionally experience smog hanging over our cities when poor air-flow and dispersal allows pollution to build up—and it is during such episodes that susceptible individuals (e.g. those with asthma, COPD or heart disease) may undergo an acute exacerbation requiring increased medication or admission to hospital. Of greater concern, however, is the inherent, modern type of pollution in today’s urban environments, which unlike the Victorian pea-souper smog, is indiscernible at ground level but manifests in chronic health effects. This ‘invisible killer’ contains nitrogen oxides, ozone (O_3_) and exceptionally small particulate matter (PM). PM_10_ and the more abundant PM_2.5_ constitute particles with diameters less than 10 and 2.5 µm, respectively—the latter being approximately 30 times less than the width of human hair. Of the modern-day air pollutants, PM has been held responsible for the majority of health effects. In urban areas, the major source is fossil fuel combustion, primarily from road transport, as well as power stations and factories. In rural and semi-urban areas of developing countries, the burning of biomass fuels on open fires or traditional stoves creates indoor concentrations of PM that far exceed those considered safe in outdoor air.

Over the last 10 years, there has been a substantial increase in findings from many research disciplines (e.g. population exposure, observational epidemiology, controlled exposure studies, animal toxicology and in vitro mechanistic work) that these modern-day ambient pollutants are not only exerting a greater impact on established health endpoints, but are also associated with a broader number of disease outcomes. The aim of this brief review article is to summarise the increased health hazards to emerge from PM air pollution research in recent years, drawing upon findings published in international projects (WHO 2012, 2013a), Health Effects Institute (HEI) research reports (HEI 2010, 2013a, b), authoritative reviews (Brook et al. 2010) and important individual publications. We will also discuss how the increased evidence base of risk relates to current public awareness and understanding of the problem. Indeed, focused education and continued evolution of sophisticated information systems have the potential to achieve a durable change in public attitude and behaviour, in a way that improves people’s health as well as the quality of the air they breathe.

## Health effects of PM air pollution

### Mortality

The ultimate effect of air pollution on public health is to bring about premature death. Epidemiological evidence first emerged from American research, which arguably began as a consequence of the 1952 air pollution episode in London. The reported associations between increased respiratory and cardiovascular mortality and acute and chronic exposures to particulate air pollution (Schwartz and Dockery 1992; Dockery et al. 1993) were subsequently confirmed outside of the USA, in many cities around the world (Katsouyanni et al. 2001; Hoek et al. 2002; Filleul et al. 2005). Of particular note, recent long-term studies show associations between PM and mortality at levels well below the current annual World health Organisation (WHO) air quality guideline level for PM_2.5_. Several updates to the Havard Six Cities Study and the study of the American Cancer Society cohort continue to cite consistent and significant associations between long-term exposure to PM_2.5_ and mortality (Lepeule et al. 2012; Krewski et al. 2009). In addition, new prospective cohorts provide additional evidence of this association, including effects observed at lower concentrations (mean 8.7 μg/m^3^; interquartile range 6.2 μg/m^3^), whilst the emerging multicity cities have confirmed previously reported increases in daily mortality (Ostro et al. 2006; Naess et al. 2007; Crouse et al. 2012; Meister et al. 2012).

We now understand that air pollution has overtaken poor sanitation and a lack of drinking water to become the main environmental cause of premature death (OECD 2014). The latest estimate from the WHO reported that in 2012, approximately 3.7 million people died from outdoor urban and rural sources (WHO 2014). The cause of deaths was broken down as follows: ischaemic heart disease (40 %), stroke (40 %); chronic obstructive pulmonary disease (COPD) (11 %), lung cancer (6 %) and acute lower respiratory infections in children (3 %). These figures are based not only on a greater understanding of the diseases caused by poor air quality, but also more accurate exposure assessment that utilises sophisticated measurement and modelling technology. Of note, the overall mortality estimate more than doubles previous ones and reveals that the vast majority of deaths stem from cardiovascular disease.

**Fig. 2:**
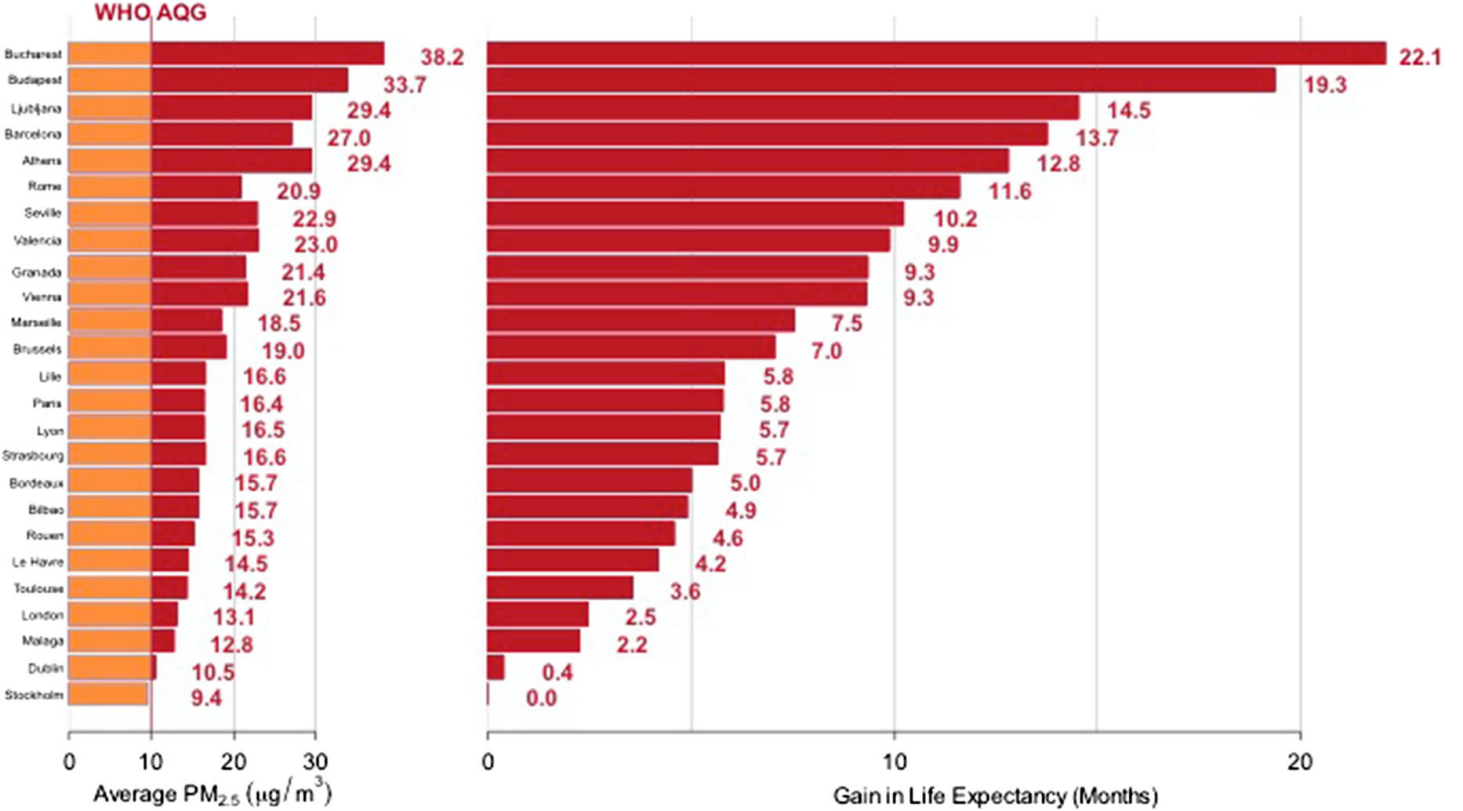
Predicted average gain in life expectancy (months) for persons 30 years of age and older in 25 Aphekom cities for a decrease in average annual level of PM_2.5_ to 10 µg/m^3^. *Source*: Aphekom project, InVS (Aphekom 2011)

By region, the largest outdoor air pollution burden is found in the low- and middle-income countries of the Western Pacific and South-East Asia, with 2.6 million linked deaths in 2012 (WHO 2014), reflecting the heavy industry and air pollution hotspots within the developing nations of these areas. However, the problem is very much a global one. Focusing on Europe, air pollution is again the biggest environment risk factor behind premature death (EEA 2014). In 2012, mortality numbers related to outdoor air pollution in the low- to middle-income, and high-income countries were estimated at 203,000 and 280,000, respectively (WHO 2014). In recent years (2010–2012) the proportion of the urban population in the 28 European Union (EU) Member States who live in areas where the EU daily limit value for PM_10_ and PM_2.5_ concentrations exceeded that was 21 and 10 %, respectively (EEA 2014). The percentage of the EU urban population exposure to PM concentrations above the WHO AQG (WHO 2006) is significantly higher, reaching 64 and 92 % for PM_10_ and PM_2.5,_ respectively (EEA 2014). Life expectancy of Europeans is reduced, on average, by about 8.6 months owing to PM_2.5_ pollution (WHO 2013b), whilst traditional health impact assessment methods used in the project Improving Knowledge and Communication for Decision-making on Air Pollution and Health in Europe (Aphekom 2011), estimates that potential exists to increase average life expectancy in the most polluted cities by approximately 22 months if PM_2.5_ concentrations were reduced to the WHO AQG annual level (Fig. 2).

In the UK, outdoor air pollution also makes a significant contribution to mortality. Current (2008) data estimate that if the effect of PM_2.5_ air pollution is considered by itself, it is responsible for at least 29,000 premature deaths, alternatively represented as an average loss of life expectancy from birth of approximately 6 months (COMEAP 2010). A more recent activity by Public Health England (PHE) has investigated the burden at a more local level in the UK, with mortality rate estimates from long-term PM_2.5_ pollution ranging from around 2.5 % in some in rural areas of Scotland and Northern Ireland, between 3 and 5 % in Wales, to over 8 % in certain London boroughs (PHE 2014). Upon comparing commonly acknowledged mortality risks, it has been estimated that a 10 µg/m^3^ reduction in ambient PM_2.5_ pollution (roughly equivalent to eradicating all anthropogenic particles) would have a larger impact on life expectancy in England and Wales than eliminating road traffic accidents or passive smoking (IOM 2006).

### Cardiopulmonary morbidity

The impact of particulate air pollution on morbidity endpoints has been subject to intense study, resulting in strong scientific consensus on the independent associations of airborne PM_2.5_ and PM_10_, with negative impacts on respiratory and cardiovascular health following both short-term and chronic exposures. Furthermore, data strongly suggest that effects have no threshold within the studied range of ambient concentrations, can occur at levels close to PM_2.5_ background concentrations and that they follow a mostly linear concentration–response function (WHO 2013a). Evidence is now well-established and particularly strong for reduced lung function, heightened severity of symptoms in individuals with asthmatics, COPD and ischaemic heart disease which includes heart attacks. We refer to previous reviews on cardiovascular effects (Brook et al. 2010) and respiratory disease (Kelly and Fussell 2011).

More recent evidence to emerge has now linked long-term exposure to PM_2.5_ to atherosclerosis—a condition that underlies many cardiovascular diseases. Indeed, the promotion and vulnerability of atherosclerotic plaques is a potential mechanism by which PM air pollution could trigger cardiovascular mortality and morbidity. In support of this, long-term exposure to PM_2.5_ concentrations, as well as proximity to traffic, is associated with preclinical markers (carotid intima media thickness [CIMT] and coronary artery calcification) of atherosclerosis (Künzli et al. 2005; Hoffmann et al. 2007; Bauer et al. 2010) and also with progression of this pathology (Künzli et al. 2010).

Emerging respiratory data now link long-term exposure to PM to childhood respiratory disease. Birth cohort studies have suggested associations between PM during pregnancy and higher respiratory need, airway inflammation and an increased susceptibility to respiratory infections (Latzin et al. 2009; Jedrychowski et al. 2013). The latest meta-analysis of 10 European birth cohorts from the ESCAPE project also provides robust evidence that post-natal PM_10_ (but notably not PM_2.5_), and traffic exposure is associated with an increased risk of pneumonia in early childhood as well as some evidence for an association with otitis media (MacIntyre et al. 2013). In a birth cohort in the Netherlands, further associations have been reported between long-term exposure to traffic-related air pollution at the birth address and both symptoms of asthma and low lung function in young children (Gehring et al. 2010; Eenhuizen et al. 2013; Molter et al. 2013). Another interesting epidemiological observation includes a possible link between chronic PM exposure during childhood and vulnerability to COPD in adulthood (Grigg 2009).

### New health outcomes

Other than the well-documented effects on respiratory and cardiovascular health, an increasing number of studies have investigated the potential of PM air pollution to negatively influence several new health outcomes. We now have evidence linking long-term exposure to PM_2.5_ with adverse birth outcomes, whilst emerging data suggest possible effects of long-term PM_2.5_ exposure on diabetes, neurodevelopment, cognitive function. The number of studies linking maternal exposure to air pollutants, including particulates, during pregnancy to various birth outcomes is steadily increasing and is of particular interest owing to the crucial time span of biological development and as such, the potential to have long-term consequences on overall health. Harmful effects have been shown for low birth weight, small for gestational age and preterm birth (Ritz and Wilhelm 2008; Sapkota et al. 2012; Proietti et al. 2013). A small number of studies have investigated traffic-related air pollution exposure at participants’ residential address as a novel risk factor for type 2 diabetes mellitus (T2DM). Although not conclusive, results suggest an association between risk of T2DM and exposure to PM (Kramer et al. 2010; Puett et al. 2011a; Coogan et al. 2012); however, evidence is stronger for NO_2_ and distance to road (Raaschou-Nielsen et al. 2013). That the deleterious effects of PM air pollution may extend to the brain have only recently been discovered and research in this area is currently limited and results inconclusive (Guxens and Sunyer 2012). For example, a study of women (68–79 years old) who lived for more than 20 years in the same residence showed a significant reduction in mild cognitive function (associated with a high risk of progression to Alzheimer’s Disease) in those who were 74 years old or younger and lived within 50 m to the next busy road with a traffic density of more than 10,000 cars per day (Ranft et al. 2009). However, no effect in cognitive function was found for PM_10_ concentrations.

### Improved air quality and improved health

**Fig. 3:**
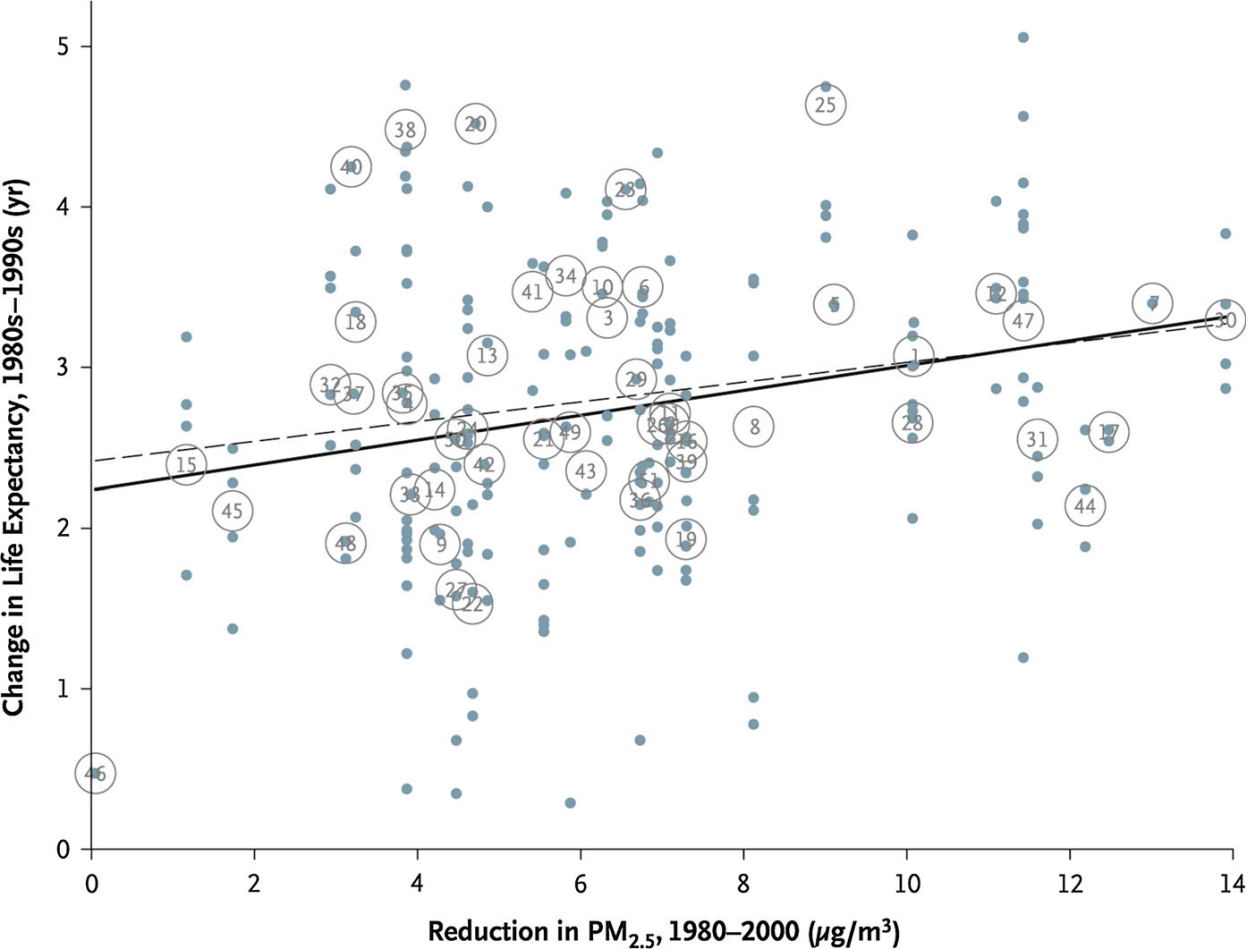
Changes in life expectancy for the 1980s–1990s plotted against reductions in PM_2.5_ concentrations for 1980–2000. *Dots and circles labelled with numbers* represent changes in population-weighted mean life expectancies at the county level and metropolitan area level, respectively. The *solid and broken lines* represent regression lines with the use of county-level and metropolitan-area-level observations, respectively. Reproduced with permission from Pope et al. (2009)

We now also have consistent evidence that a reduction in the level of particulate pollution following a sustained intervention (mainly regulatory actions) is associated with improvements in public health. In the USA, Pope et al. (2009) used data from the 51 cities from the American Cancer Society study for which long-term PM_2.5_ data are available. It was reported that after adjustment for changes in other risk factors, the reduction in PM_2.5_ concentration between 1980 and 2000 was strongly associated with 2.7 year overall increases in life expectancy that occurred during that period (Fig. 3). Evidence has also been demonstrated in the Swiss Study on Air Pollution and Lung Diseases in Adults (SAPALIDIA) that assessed lung diseases in adults in eight communities in 1991 and again in 2002—a period when the annual average PM_10_ concentration decreased by 5–6 µg/m^3^. This reduction in particle levels was associated with attenuation in the annual rate of decline of lung function (Downs et al. 2007). Using the same cohort, Schindler et al. (2009) reported that fewer reports of regular cough, chronic cough or phlegm, and wheezing and breathlessness could also be attributed to the observed decrease in PM_10_. In a separate Swiss investigation following children from nine Swiss communities between 1992 and 2001, declining concentrations in ambient PM_10_ was associated with improved respiratory health (reduced incidence of chronic cough, bronchitis, common cold, nocturnal dry cough and conjunctivitis symptoms; Bayer-Oglesby et al. 2005). The results suggest that health improvements can be expected to appear almost immediately and can be seen following almost any decrease in the concentration of PM (for example, the observed beneficial effects in respiratory health of the Swiss children occurred following relatively small changes of rather moderate air pollution levels) enormously strengthens the argument for optimal air quality management.

## Differential toxicity of PM

Epidemiological and toxicological research findings have shown that PM mass (PM_2.5_ and PM_10_) comprises fractions and sources with varying types and degrees of health effects (Kelly and Fussell 2012). The subject of relative toxicity represents one of the most challenging areas of environmental health research in that PM is not a single entity. It is a complex, heterogeneous mixture that can exist as solids or liquids. These particles vary not only in chemical composition, mass, size (few nanometres to tens of micrometres), number, shape and surface area, but also source, reactivity, solubility and reactivity. In London, particulate pollution is predominantly diesel exhaust particles (DEPs) mixed with resuspended particles of tyre rubber and brake dust (Yanosky et al. 2012). This compares with combinations of traffic-derived PM and desert sand in parts of Ghana, biomass smoke Ethiopia and soot from coal fired power stations in the eastern provinces of China. All of the many characteristics of PM have the potential to influence the toxicity of ambient PM. Current knowledge does not, however, allow individual characteristics or sources to be definitely identified as being closely related to specific health effects and likewise, no specific source, component or size category can be excluded as having no adverse effects (EPA 2009; WHO 2013a; HEI 2013a). Rather, the capability of PM to induce disease may be the result of multiple components acting on different physiological mechanisms.

Below is a brief overview of current evidence on the contribution to adverse health effects played by chemical constituents (black carbon [BC], organic carbon [OC], inorganic secondary aerosols), size (coarse PM, ultrafine particles [UFP) and source (road transport). The findings discussed have arisen from the WHO REVIHAAP Project (Review of Evidence on Health Aspects of Air Pollution) (WHO 2013a), together with other recent critical reviews and systematic research efforts on the subject (WHO 2012; HEI 2010, 2013a, b).

### Black carbon

In addition to the WHO REVIHAAP project (WHO 2013a), the health effects of BC particles have also undergone a recent systematic review by the WHO Regional Office for Europe (WHO 2012). These initiatives have confirmed that sufficient epidemiological evidence exists to link short-term (daily) variations in BC particles with all-cause and cardiovascular mortality and cardiopulmonary hospital admissions. Evidence is also conclusive that long-term (annual) BC exposure is associated with all-cause and cardiopulmonary mortality. Although distinct mechanistic effects have not been identified from toxicological studies, suggesting that BC may not be a direct toxic component of fine PM, it is hypothesised that these particles may operate as a universal carrier of combustion-derived chemicals (semi-volatile organic fractions, transition metals) of varying toxicity to not only the lungs, but to systemic circulation and beyond. Furthermore, in that short-term studies show that health effect associations with BC were more robust than those with PM_2.5_ or PM_10_, although BC particles may not constitute a causal agent, it is the opinion that they may well serve as a better indicator of harmful particulate substances (e.g. organics) from primary traffic-related combustion particles compared to undifferentiated PM mass (WHO 2012).

### Organic carbon

OC is a very complex and heterogenous mixture of primary and secondary organic aerosols and owing to the common combustion source, can co-exist with BC. As a consequence, it is a huge challenge to identify the potential toxicity of specific OC constituents and in fact evidence is currently insufficient to distinguish between the toxicity of primary and secondary organic aerosols. Studies are, however, generating increasing amounts of data on associations between total organic carbon and a variety of health effects including short-term perturbations in both respiratory (Kim et al. 2008; Hildebrandt et al. 2009) and cardiovascular (Delfino et al. 2010; Ito et al. 2011; Kim et al. 2012; Son et al. 2012; Zanobetti and Schwartz 2009) endpoints. Ostro et al. (2010) has also observed associations between long-term exposure to organic carbon and both ischaemic heart disease and pulmonary mortality.

### Inorganic secondary aerosols

Epidemiological evidence continues to accumulate on the short-term effects of sulphate on cardiovascular mortality as well as both respiratory and cardiovascular hospital admissions (Ito et al. 2011; Kim et al. 2012). Data have also emerged on associations between daily increments in ambient sulphate and physiological changes to the cardiovasculature, namely ventricular arrhythmias (Anderson et al. 2010) and markets of endothelial dysfunction (Bind et al. 2012). The HEI’s comprehensive National Particle Component (NPACT) initiative also identified significant associations in the epidemiological studies between secondary inorganic sulphate and health effects and moreover, these were backed up by complimentary findings in the toxicological element of the project (HEI 2013a). Historically, toxicological evidence has been seemingly consistent that the components of inorganic secondary aerosols (ammonium, sulphates or nitrates) pose little threat, but uncertainties do exist. For example, the cations (metals, hydrogen) associated with sulphates/nitrates and/or other absorbed components (metals, organic particles) may have an underlying toxic role or else secondary inorganic components may influence bioavailability and as a consequence, toxicity of other particulate components (Oakes et al. 2012).

### Coarse PM

Accumulating epidemiological evidence suggests that short-term exposures to coarse particles (between 2.5 and 10 μm) are associated with effects on adverse cardiovascular health, respiratory endpoints health, including premature mortality (Peng et al. 2008; Atkinson et al. 2010; Mann et al. 2010; Meister et al. 2012; Qiu et al. 2012). Overall opinions made by various systematic reviews and assessments are variable as to whether such effect estimates are higher or lower than those for fine PM (Brunekreef and Forsberg 2005; EPA 2009). Investigations into long-term effects of coarse PM are fewer and have reported no or limited evidence that this size fraction has an effect on mortality or cardiovascular health (Puett et al. 2009, 2011b). Toxicological studies comparing coarse and fine PM have reported that coarse particles can be as toxic as PM_2.5_ on a mass basis (Graff et al. 2009; Wegesser et al. 2009). Data, however, are not only scarce, but also difficult to interpret owing differences in the inhalability and deposition efficiency of these size fractions.

### Ultrafine particles

UFPs (smaller than 0.1 μm) have many unique properties that have led scientists to hypothesise that this size fraction may have specific or enhanced toxicity relative to fine (PM_2.5_) or coarse PM. Apart from the relationship between particle diameter and penetration within the lung and to extrapulmonary sites, on a mass basis, smaller particles have a much greater surface area and with that, a high capacity to adsorb toxic chemicals. In addition, the finer the particles, the greater the likelihood of penetration to indoor environments, being suspended in the atmosphere for longer periods and being transported over large distances. As a consequence, a substantial body of literature has now been published on the mechanisms of UFP toxicity and adverse effects in animals and in humans, including a recent HEI review (HEI 2013b). Epidemiological data, however, are still limited and provide suggestive rather than strong and consistent evidence of adverse effects of UFPs [reviewed by Rückerl et al. (2011), Weichenthal (2012)]. The HEI review noted that “The current evidence does not support a conclusion that exposure to UFPs alone can account in substantial ways for the adverse effects of PM_2.5_” (HEI 2013b). Toxicological studies have certainly advanced our understanding of the action of UFPs, showing the potential of this size fraction to adopt differential patterns of deposition, clearance and translocation (Kreyling et al. 2010).

### Source

Many PM pollution sources, namely coal combustion, shipping, power generation, the metal industry, biomass combustion, desert dust episodes and road transport have been associated with different types of health effects (EPA 2009; WHO 2013a). Of these, the main source of urban pollution—road transport—is also the source associated with the most serious health outcomes. A critical review of the literature on the health effects of traffic-related air pollution concluded that sufficient evidence had accumulated to support a causal relationship between exposure to traffic-related air pollution and exacerbation of asthma. Further evidence was found to be *suggestive* of a causal relationship with onset of childhood asthma, non-asthma respiratory symptoms, impaired lung function, total and cardiovascular mortality, and cardiovascular morbidity (HEI 2010).

The PM components from road traffic include engine emissions, comprising largely of EC and OC, plus non-exhaust sources that are often characterised by elevated concentrations of transition metals (brake wear [copper, antimony], tyre abrasion [zinc], dust from road surfaces [iron]). The largest single source is derived from diesel exhaust (DE). Indeed, owing to the increased domestic market penetration of diesel engines, the fuel powering the majority of our buses and taxis in many industrialised countries and the fact that they generate up to 100 times more particles than comparable gasoline engines with 3-way catalytic convertors (Quality of urban air review group 1996), diesel exhaust particles (DEPs) contribute significantly to the air shed in many of the world’s largest cities. DEPs have also been shown to have substantial toxicological capacity, facilitated by the size (80 % of DEPs have an aerodynamic diameter of <1 µm) of such particles as well as their surface chemistry characteristics. For instance, DEPs have a highly adsorptive carbon core that act as a vector for the delivery, deep into the lung, of redox active metals, polyaromatic hydrocarbons and quinones. In addition to traffic density per se, it is not surprising therefore that the greatest health impacts appear to be associated with proximity to roads carrying a high proportion of diesel powered heavy and light good vehicles (Janssen et al. 2003; Gowers et al. 2012). In 2012, the International Agency for Research on Cancer (IARC) classified particulates in diesel fumes as carcinogenic to humans based on sufficient evidence that it is linked to an increased risk of lung cancer, as well as limited evidence linking it to an increased risk of bladder cancer (IARC 2012). Although most studies into the toxicity and health consequences of roadside PM have focused on DEPs, the non-exhaust sources are attracting interest and deservedly so (van der Gon et al. 2013). Whilst contributions from brake/tyre wear and road surface abrasion in the wake of passing traffic will become more important with progressive reductions in exhaust emissions, their potential to elicit health effects is largely ignored at the regulatory level despite links with cardiopulmonary toxicity (Gasser et al. 2009; Gottipolu et al. 2008; Mantecca et al. 2009; Riediker et al. 2004).

## Mechanisms of PM toxicity

**Fig. 4:**
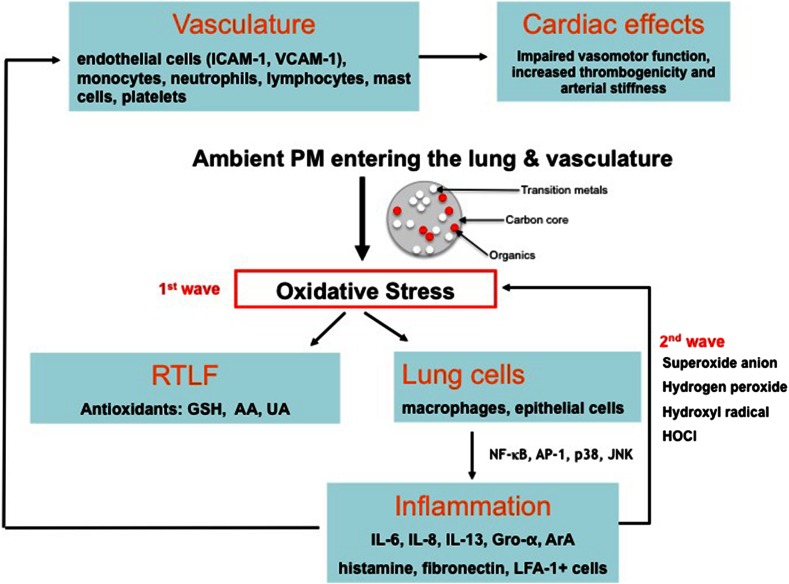
Biological pathways linking PM exposure with oxidative and inflammatory pathways in the lung and cardiovasculature

The well-established evidence that PM pollution contributes to an array of health outcomes has resulted in an enormous research effort to understand the underlying biological basis of toxicity by identifying mechanistic pathways. Although there remains much to be understood, our appreciation of the physiological effects and plausible biological mechanisms that link short- and long-term PM_2.5_ exposure with mortality and morbidity has evolved rapidly and continues to do so. For example, in investigating subclinical physiological changes, epidemiological research has reported variations in cardiovascular biomarkers of systemic inflammation such as C-reactive protein and fibrinogen—subtle responses that have been consistently linked to subsequent cardiovascular disease and death (Brook et al. 2010). A particularly powerful tool to study mechanistic pathways and physiological endpoints related to adverse effects following DEP exposure is the use of controlled exposure studies in healthy volunteers (Salvi et al. 1999, 2000; Mudway et al. 2004; Pourazar et al. 2004, 2005; Mills et al. 2005; Behndig et al. 2006; Peretz et al. 2007; Tornqvist et al. 2007; Lucking et al. 2008; Lundback et al. 2009) as well as mild asthmatics (Stenfors et al. 2004) and individuals with stable coronary heart disease (Mills et al. 2007). Carefully characterised and environmentally relevant DE exposure experiments, combined with cardiovascular measurements, BAL and bronchial biopsy have revealed well-defined systemic, pulmonary and cardiac responses involving a variety of cellular and molecular perturbations. It is probable that associations between the various constituents of PM and health effects are the result of multiple, complex and interdependent mechanistic pathways acting on different physiological mechanisms. Current evidence does, however, support a chain of events involving pollution-induced pulmonary and systemic oxidative stress and inflammation, translocation of particle constituents and an associated risk of vascular dysfunction, atherosclerosis, altered cardiac autonomic function and ischaemic cardiovascular and obstructive pulmonary diseases (Kelly and Fussell 2015; Fig. 4).

Since oxidative stress is widely believed to play a key role in the harmful effects of a range of particles at the cellular level, the oxidative potential (OP) of PM (their capacity to cause damaging oxidative reactions) is regarded as an attractive exposure metric in our bid to identify the toxic components and sources within an ambient PM mix (Ayres et al. 2008; HEI 2010). The OP of ambient PM collected at busy roadside sites is clearly enhanced relative to urban background and rural sites, and this appears to be associated with an enrichment of metals (copper, barium) linked with abrasion and brake wear processes (Godri et al. 2011; Kelly et al. 2011; Boogaard et al. 2012). Concern surrounding these findings is well founded in that they highlight a toxic contribution by non-exhaust pollutants and as such, by a currently unregulated source. Oxidative stress is linked to several DNA lesions and the formation of bulky adducts—mechanisms by which traffic-related pollution could elicit mutagenesis and in turn cause cancer (Loeb 2001). Of the biomarkers of oxidatively damaged DNA, urinary excretion of 8-oxo-7,8-dihydro-2-deoxyguanosine (8oxodG) has now been validated to evaluate the pro-oxidant effects of vehicle exhaust emissions on the DNA of exposed subjects (Barbato et al. 2010). DNA adduct levels in non-smoking workers have been found to reflect average levels of exposure to PM_10_ in high-traffic urban areas (Palli et al. 2008) and were also reported to be increased in cord blood after maternal exposures to traffic-related air pollution (Pedersen et al. 2009)—the latter demonstrating the potential for transplacental environmental exposures to induce DNA damage early in newborns and with that, increased risk for adverse effects later in life.

## Public awareness and education

That poor air quality can have such a significant impact on human health is undisputed, and the previous sections have drawn upon research conducted over recent years that supports the notion that risks are increasing as new hazards emerge. How then does this translate to public awareness of the problem? The general consensus is that society would benefit from being better engaged and educated about the complex relationship between air quality and ill health (Kelly et al. 2012). If people are aware of variations in the quality of the air they breathe, the effect of pollutants on health as well as concentrations likely to cause adverse effects and actions to curtail pollution, there follows a greater likelihood of motivating changes in both individual behaviour and public policy. In turn, such awareness has the potential to create a cleaner environment and a healthier population.

Studies and initiatives examining public awareness and understanding in this area have yielded mixed results, with some acknowledging a significant amount of concern within the public over poor air quality, an awareness of air quality warnings, and a positive relationship between alerts and a change in outdoor activities (DEFRA 2002; Wen et al. 2009; McDermott et al. 2006). In fact, following findings that air quality warnings associated with ground level O_3_ do have a significant impact on attendance at outdoor facilities in Southern California, Neidell and Kinney (2010) suggested that ambient air quality measurements from monitors may not reflect personal exposure if individuals intentionally limit their exposure in response poor air quality. Bell et al. (2004) have also hypothesised that deliberate avoidance in time spent outdoors could contribute to the considerable heterogeneity in O_3_-induced mortality observed across US communities. Other research has concluded that both awareness of the links between air pollution and ill health and an understanding of air quality information are lacking amongst the public (Bickerstaff and Walker 2001; Semenza et al. 2008; COMEAP 2011). In 2013, the European Commission (EC) conducted a flash Eurobarometer to gain a greater insight into the views of the European public on matters of air quality and air pollution (EC 2013). Six out of ten Europeans responded that they did not feel informed about air quality issues in their country. When asked how serious they considered a range of air quality related problems to be in their country, responses for respiratory disorders, cardiovascular diseases and asthma/allergy were 87, 92 and 87 % respectively.

### Factors determining awareness

Other than the availability of sufficient information that will be covered in the following section, factors governing how aware individuals are about the quality of their air and potential repercussions for their health are likely to include understanding, perception and a vested interest. Individuals may choose not to concern themselves about air quality owing to a poor understanding of what is undoubtedly a complex science. Unlike other environmental risks that are routinely communicated such as UV and heat, overall air quality encompasses several primary pollutants as well as secondary products owing to atmospheric transformation. Rural areas for example are very often considered safe places to escape from pollution. However, at times, O_3_ concentrations can be as high or greater than urban locations owing to the presence of lower concentrations of nitrogen oxides to sequester rural O_3_. A lack of vested interest in the topic is also possible amongst ‘healthy’ people, less likely to have any personal experience of the benefits that lessoning pollution and/or increasing medication may bring. Indeed, where research has indicated that individuals are aware of air quality warnings and take responsive actions, larger responses were observed for more susceptible groups or carers thereof (McDermott et al. 2006; Wen et al. 2009). Within a cross-sectional study of 33,888 adults participating in the 2005 Behavioral Risk Factor Surveillance System, 31 % with asthma versus 16 % without changed outdoor activity in response to media alerts (Wen et al. 2009). Perception is another factor influencing the public understanding of the importance of healthy air, as attitudes and behaviour can be driven by a person’s immediate locality and own understanding rather than accurate data generated by monitoring sites and communicated via an advisory service (Shooter and Brimblecombe 2009). Several studies have investigated the relationship between perceived and measured outdoor air quality provided by monitoring stations and whilst some studies found a significant association between the perception of air quality and specific air pollutants (Atari et al. 2009), others have found little or no association (Rotko et al. 2002). Of relevance, Semenza et al. (2008) not only reported a low (10–15 %) level of behavioural change during an air pollution episode, but that the personal perception of poor air quality rather than the advisory service, drove the response. Some epidemiological researchers have also indicated that self-reported health status is associated with perceived air pollution rather than measured air pollution (Lercher et al. 1995; Yen et al. 2006; Piro et al. 2008).

### Information services

**Fig. 5:**
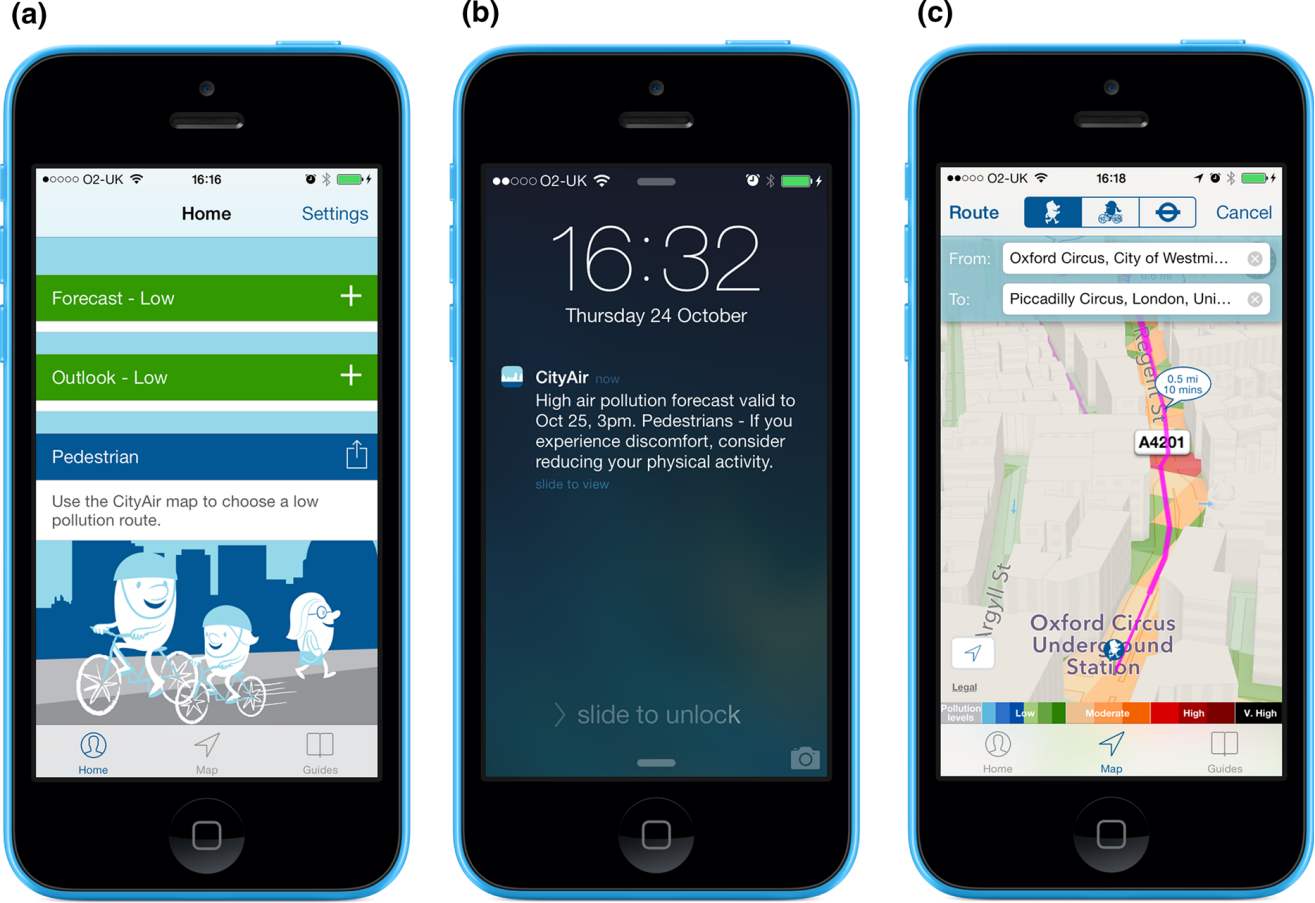
City Air iPhone app. **a** Advice tailored to specific user groups. **b** Notification alert when pollution levels change. **c** 3-D low pollution journey planners

Public awareness is fundamentally dependent upon optimal air pollution monitoring, forecasting and reporting. Many countries have air quality monitoring networks that are structured around a particular country’s regulatory obligation to report monitored air quality data and modelled predictions (Kelly et al. 2012). Output from measured concentrations of pollutants, air quality modelling systems and meteorological data are also processed to create a national air quality index (AQI). Again in line with national legislation, an AQI communicates pollution levels and health effects likely to be experienced on the day described by the index or days soon afterwards (i.e. the short term; Table 1). These data are used by the public and organisations (health services and governments) to reduce the health impacts of predicted air pollution. For example, people susceptible to high levels of pollution may be prompted to take actions (reduce exposure and/or increase use of inhaled reliever medication) to reduce their symptoms, and the general public may be encouraged to use public rather than private transport during periods of poor air quality. Another information tool is provided by accessible air pollution alert services that provide real-time data and proactively alert registered users of impending pollution events via a computer/tablet (websites, email, social media) or phone (texts, apps) (London Air Quality Network; City of London). These are becoming increasingly informative and engaging, allowing people to sign up to specific user groups (e.g. cyclist, jogger, business, at risk) and receive notifications when pollution exceeds concentrations at a site(s) of their choice. These services also offer tailored advice on how specific groups can reduce emissions by for example, providing low pollution journey planners to reduce exposure (Fig. 5).

**Table 1:** Health advice for the general population to accompany the UK AQI

Air pollution banding	Value	Accompanying health messages for at-risk groups and the general population	
		At-risk individuals^a^	General population
Low	1–3	*Enjoy* your usual outdoor activities	*Enjoy* your usual outdoor activities
Moderate	4–6	Adults and children with lung problems, and adults with heart problems, *who experience symptoms*, should *consider reducing* strenuous physical activity, particularly outdoors	*Enjoy* your usual outdoor activities
High	7–9	Adults and children with lung problems, and adults with heart problems, should *reduce* strenuous physical exertion, particularly outdoors, and particularly if they experience symptoms. People with asthma may find they need to use their reliever inhaler more often. Older people should also *reduce* physical exertion	Anyone experiencing discomfort such as sore eyes, cough or sore throat should *consider reducing* activity, particularly outdoors
Very high	10	Adults and children with lung problems, adults with heart problems, and older people, should *avoid* strenuous physical activity. People with asthma may find they need to use their reliever inhaler more often	*Reduce* physical exertion, particularly outdoors, especially if you experience symptoms such as cough or sore throat

^a^Adults and children with heart or lung problems are at greater risk of symptoms

### New developments

Whilst monitoring, forecasting and reporting of air quality have become increasingly sophisticated and accurate, the future use of more individualised exposure measurements holds a great deal more potential. Air pollution levels can vary dramatically over short distances and time scales and in addition people’s daily mobility and activities will result in variability in exposure and inhalation. As such, AQIs and alert systems sourced by fixed site monitoring stations are always going to be limited by location, spacing and density. Up until recently, the use of personal pollution monitors was primarily limited to industries associated with high occupational exposures and researchers assessing individual exposures in vulnerable groups such as cyclists (Nwokoro et al. 2012) and asthmatic children (Spira-Cohen et al. 2011). Now we are witnessing an emerging role for inexpensive, portable, easy-to-use personal monitoring devices (Austen 2015). Although the quality of information generated by such sensors is not currently robust enough to compliment data for official monitoring networks, there is undoubtedly a need for more dynamic measures of time-activity patterns in relation to exposures. In an initiative to better understand in real time the impacts of harmful air pollutants, the US Environmental Protection Agency awarded a $100,000 prize to designers of a low-cost wearable breathing analysis tool that calculates the amount of polluted air a person breathes and transmits the data to any Bluetooth-enabled device such as a mobile phone (EPA 2013). Smart phone technology, integrated with low-cost air quality sensors, also has the potential to produce dynamic, temporally and spatially more precise exposure measures for the mass population. Added to their ubiquitous technology, the penetration of these phones is unrivalled in demographics, geographic coverage, acceptance and presence in everyday life (Pratt et al. 2012). This opens up new possibilities in the communication of individual exposure and activity data, tailored to locations where people commute and reside. In the environmental research setting, novel smartphone-based software that records people’s movements and physical activity levels in the urban environment and is integrated with spatial–temporal maps of air pollution is already being developed to enhance large-scale air pollution exposure data collection in a cost-effective, accurate unobtrusive way (de Nazelle et al. 2013).

## Discussion

Despite past improvements in air quality, very large parts of the population in urban areas breathe air that does not meet European standards let alone WHO Air Quality Guidelines. It should not be surprising therefore that health effects of PM—one of the pollutants deemed most dangerous to health—are well documented. Airborne PM has been the focus of extensive research and debate around the world for several decades and as a consequence, the evidence base for the association between short- and long-term exposure to PM and cardiopulmonary mortality and morbidity has become much larger and broader. DEPs are now classified as carcinogenic, and an increasing number of studies are investigating the potential for particulate air pollution to negatively influence birth outcomes, diabetes, neurodevelopment and cognitive function. We now also appreciate that there is no evidence of a safe level of exposure or a threshold below which no adverse health effects occur, with recent long-term studies are showing associations between PM and mortality at levels well below the current annual WHO air quality guideline level for PM_2.5_. Correspondingly, reductions in population exposure to air pollution expressed as annual average PM_2.5_ or PM_10_ have appreciable benefits in terms of increased life expectancy and improvements to respiratory health.

Having firmly established associations between ambient PM and adverse health effects, there has been an enormous effort to identify what it is in ambient PM that affects health—information that in turn will inform policy makers how best to legislate for cleaner air. The topic of relative toxicity has been the subject of several critical reviews over recent years but despite this the general conclusion remains that the current database of experimental and epidemiologic studies precludes individual characteristics or sources to be definitely identified as critical for toxicity. A better understanding of exposure and health effects plus further progress in comparing and synthesising data from existing studies is therefore needed before concluding that additional indicators (be they BC or UFPs) have a role in protecting public health more effectively than the targeting total PM mass. Another challenge has been to unravel the underlying biological basis of toxicity by identifying pathways that ultimately link pollution-induced pulmonary and systemic oxidative stress with an associated risk of cardiovascular and obstructive pulmonary diseases.

Evidence has emerged that (a) the burden of ambient PM pollution on health is significant at relatively low concentrations, (b) there is no safe lower limit and (c) effects follow a mostly linear concentration–response function, suggesting that public health benefits will result from any reduction in concentrations. As has been advocated many times before, interventions to reduce levels of particulate pollution require a concerted action by a host of sectors with a vested interest in air quality management (environment, transport, energy, health, housing) at regional, national and international levels. The significant toll of ill health brought about by traffic-related particulates means that forward-looking and integrated transport policies are critical for the improvement of urban environments. Traffic must be reduced and we must ensure a cleaner and greener element to what remains on the road. This can be achieved through an expansion of low emission zones, investment in clean and affordable public transport and incentives for its use, a move back from diesel to petrol or at least a ban on all highly polluting diesel vehicles, lowering speed limits and enhancing cycle routes.

Another intervention in moving towards a cleaner and healthier environment necessitates behavioural changes by the public, which in turn requires continued education and optimal communication. Engagement must be blatant and put in the context of other public health risks such as passive smoking, it must also utilise compelling messages such as premature death. In an ideal world, people, and especially susceptible individuals, should be aware of their air quality by regularly checking the AQI or targeted notifications for real-time data before going to work, school or to pursue leisure activities, enabling them to take action in the event of increased pollution. Improving air quality is a considerable but not an intractable challenge. Translating the correct scientific evidence into bold, realistic and effective policies undisputedly has the potential to reduce air pollution so that it no longer poses a damaging and costly toll on public health.
